# How Point-of-Care Ultrasound Led to a Diagnosis of May-Thurner Syndrome

**DOI:** 10.24908/pocus.v6i2.15105

**Published:** 2021-11-23

**Authors:** Daniel L Belkin, Mitchell D Belkin, Maedeh Ashrafi, Charan Vegivinti, Yung-Hsien Wang, Leonidas Palaiodimos

**Affiliations:** 1 Albert Einstein College of Medicine Bronx, New York, 10461; 2 University of Maryland School of Medicine Baltimore, Maryland, 21201; 3 Department of Medicine, Jacobi Medical Center, NYC H+H Bronx, New York, 10461

**Keywords:** deep vein thrombosis, DVT, point-of-care ultrasound, POCUS, May- Thurner syndrome, Vascular, thrombectomy

## Abstract

A 65-year-old man with a history of a left-sided inguinal hernia presented with three days of left-sided groin pain worsened with exertion and fatigue. The patient was afebrile but tachycardic, and physical examination revealed a tender, erythematous immobile bulge in his left groin. Laboratory studies revealed leukocytosis. Lymphadenopathy secondary to infectious or inflammatory etiology was suspected. However, point-of-care ultrasound (POCUS) identified extensive deep vein thrombosis (DVT) of the lower left limb. Follow-up imaging revealed this to be secondary to May-Thurner syndrome, a mechanical compression of an iliocaval vein against the lumbar vertebrae by a common iliac artery. This report demonstrates how POCUS can be used to identify lower extremity DVT, thereby expediting diagnosis and treatment and potentially preventing complications.

## Introduction

May-Thurner syndrome (MTS) is the mechanical compression of an iliocaval vein, most commonly the left iliac vein, against the lumbar vertebrae by the arterial system, most commonly the right common iliac artery . Although it may be asymptomatic, this uncommon condition places individuals at risk for extensive deep vein thrombosis (DVT) of the left lower limb. Our case demonstrates how clinicians utilized point-of-care ultrasound (POCUS) to diagnose extensive left lower extremity DVT, thereby expediting the diagnosis and treatment of MTS.

## Case presentation

A 65-year-old man with a history of active tobacco use (40 pack-year) and left-sided inguinal hernia presented to the emergency room with fatigue and anorexia associated with left-sided groin pain that was aggravated by exertion. The symptoms started three days prior to presentation. The patient denied any other symptoms in open- and closed-ended questions. He reported mild intermittent left-sided groin pain attributable to his known inguinal hernia. However, he denied similar symptoms in the past. He did not take any medications, denied sexual activity for the past year, and denied toxic habits other than tobacco use. He worked as a building superintendent and described himself as active and healthy. His body mass index was 24 kg/m^2^.

On presentation, the patient was afebrile, heart rate was 111 beats per minute, respiratory rate was 17, blood pressure was 130/80 mm Hg, and SpO_2_ was 97% on ambient air. The physical examination was remarkable only for a small erythematous, palpable, tender, well-defined, immobile bulge in his left groin surrounded by mild erythema, which spread to his left inner thigh in the first two days of hospital stay. The rest of the physical examination was unremarkable. The laboratory work-up was remarkable for neutrophilic leukocytosis (WBC 14.83/nL) and significantly elevated C-reactive protein (CRP 230.3 mg/L). Hemoglobin, platelet count, basic coagulation panel, and comprehensive metabolic panel were unremarkable.

**Figure 1:**
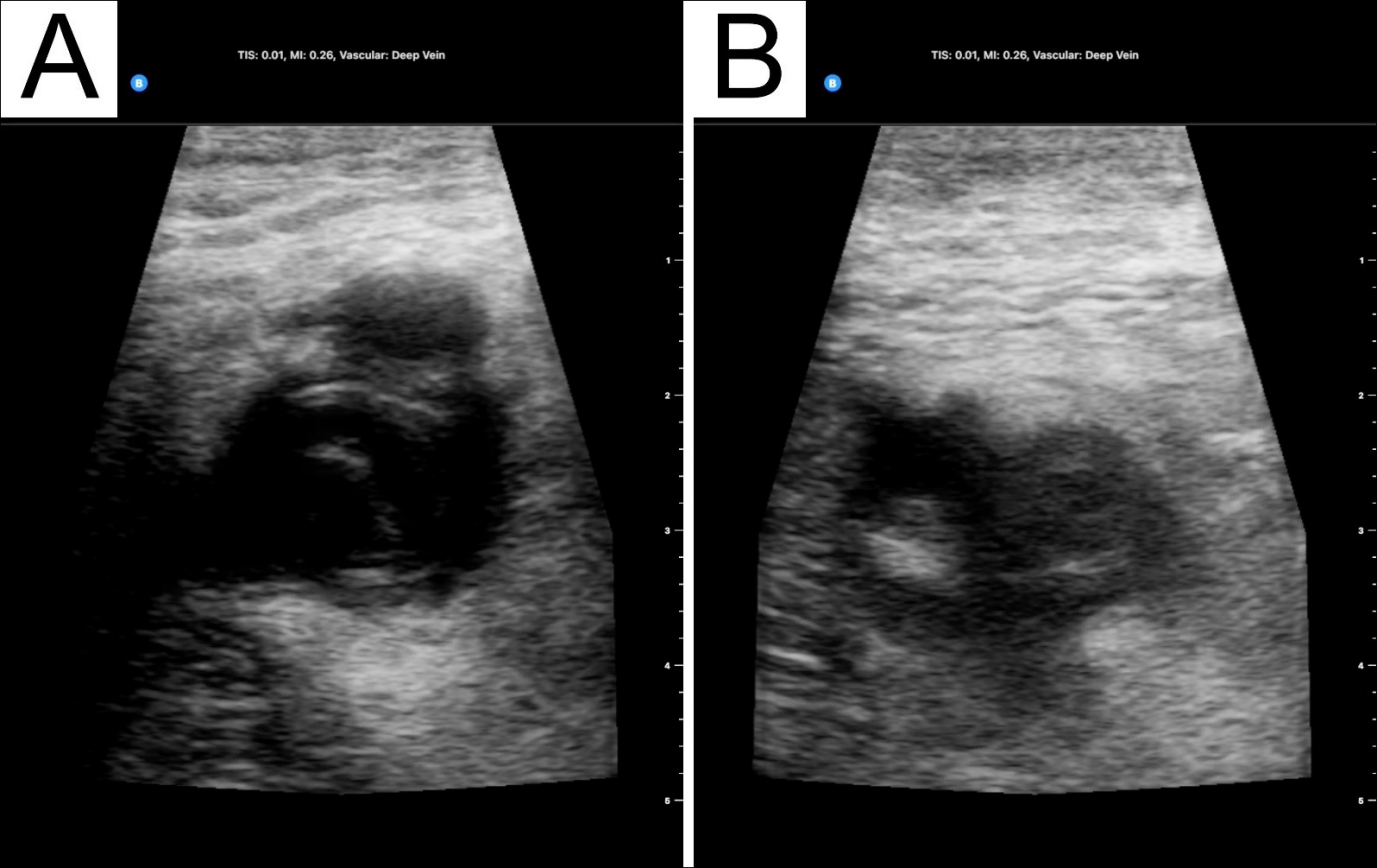
(A) A distended left Common Femoral Vein seen on point-of-care ultrasound (POCUS). (B) The same vein proves to be non-compressible when pressure was applied.

At this point, the admitting medicine team was considering an infectious or inflammatory etiology and believed the palpable lesion in the left groin to be secondary to lymphadenopathy rather than an inguinal hernia as the patient believed. A point-of-care ultrasound examination was performed to visualize the enlarged lymph nodes. Surprisingly, a large echogenic thrombus was seen extending from the common femoral vein (CFV) to the mid-thigh. The CFV and femoral vein (FV) were distended and non-compressible (Figure 1, online Video S1). 

**Figure 2:**
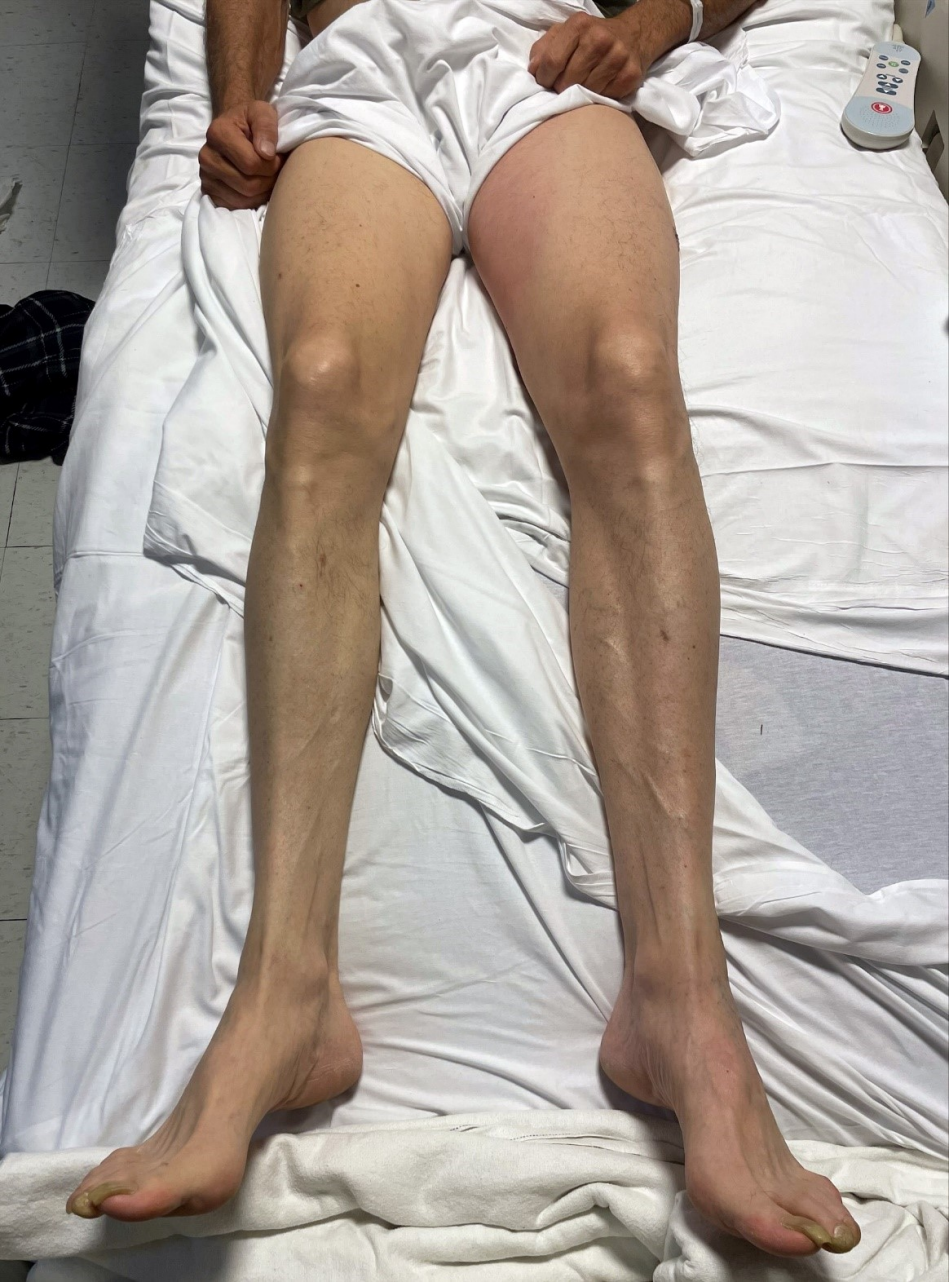
Picture of the patient’s lower extremities showing no significant edema, erythema or asymmetry.

This finding came as a surprise as no lower extremity edema was noted on physical exam (Figure 2). Up until this visualization with point-of-care ultrasound, DVT had not been in the working differential diagnosis.

**Figure 3:**
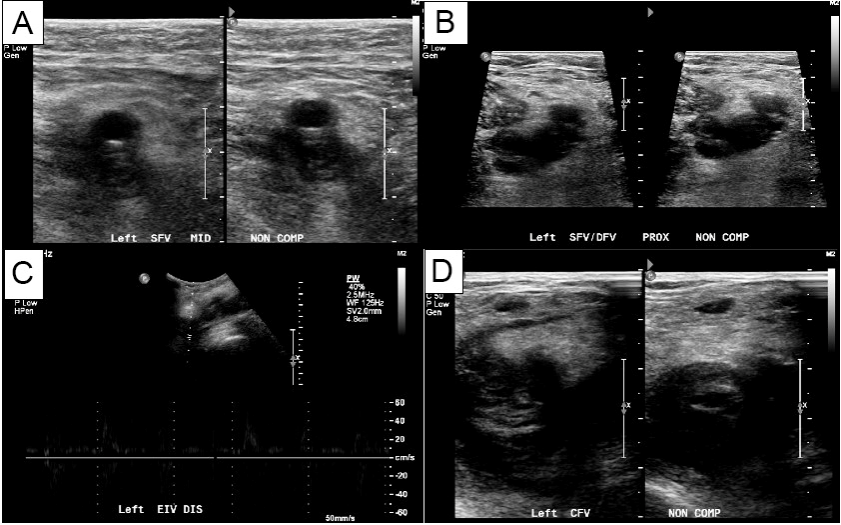
A vascular-performed duplex ultrasound revealed acute non-compressible deep vein thrombosis in the left lower extremity involving (A) the left superficial femoral vein; (B) the left deep femoral vein; (C) the left external iliac vein; (D) the left common femoral vein.

A vascular lab performed duplex ultrasound was ordered to confirm our bedside findings and revealed extensive DVT in the left lower extremity including the distal external iliac vein, CFV, proximal deep femoral vein, proximal-to-distal FV, as well as acute superficial venous thrombosis in the great saphenous vein at the level of the saphenous-femoral junction and proximal thigh (Figure 3). 

**Figure 4:**
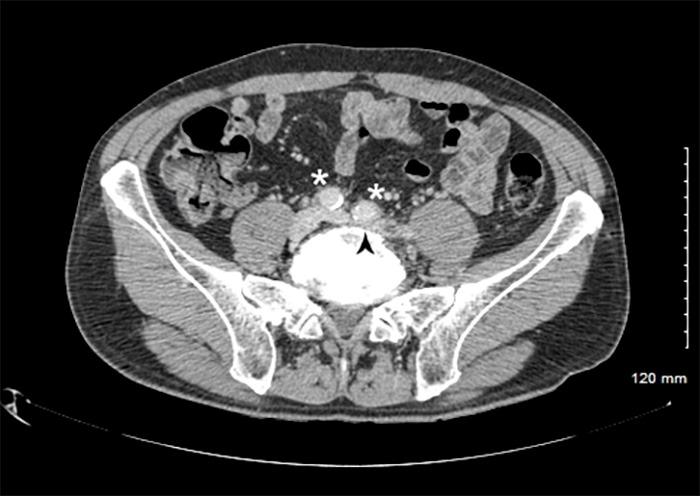
Contrast-enhanced CT showed that the left iliac vein (arrowhead) was compressed by the left and right common iliac arteries (asterisk), consistent with a diagnosis of May-Thurner syndrome.

In the meantime, d-dimer concentrations were found to be 1840 ng/mL and therapeutic anticoagulation with Enoxaparin 1 mg/kg every 12 hours was initiated. Computed tomography (CT) of the chest, abdomen, and pelvis with contrast revealed findings consistent with May-Thurner syndrome, with the left and right common iliac arteries compressing the left iliac vein proximal to the clot (Figure 4). 

Vascular surgery was consulted and the patient underwent localized thrombolysis, thrombectomy and stent placement in the left iliac vein. The patient tolerated the procedure and did not have any complications. His symptoms, including fatigue and anorexia, gradually resolved. CRP concentrations declined to 115.4 mg/L, and the patient was discharged on post-op day 3 on Aspirin, Clopidogrel, and Apixaban with vascular surgery follow-up and age-appropriate cancer screening including colonoscopy. Of note, CT of the chest, abdomen, and pelvis did not reveal any evidence of malignancy, and prostatic-specific antigen (PSA) concentration was unremarkable. Thrombophilia work-up was not indicated given the patient's age, the absence of a history of thromboembolic episodes, and the detection of an anatomic risk factor for thrombosis, MTS. 

## Discussion

May-Thurner syndrome is estimated to cause 2-5% of all DVTs 1. MTS is most common in patients between 18 and 50 years old, with women being five times more likely than men to suffer from the condition 2. MTS patients that have additional risk factors such as recent surgery, pregnancy, oral contraceptive pill use, and malignancy are more likely to form blood clots and develop DVT 2. Furthermore, patients with asymptomatic MTS who also have hypercoagulable disorders, infection, or dehydration are more likely to develop a DVT 2. 

The gold standard to diagnose MTS is CT venography with transvenous pressure measurements. However, this invasive procedure entails potential complications, including phlebitis; thus, other imaging modalities are first line, including ultrasound doppler, which is an easy and inexpensive screening modality 1, 3. On ultrasound doppler, MTS can be diagnosed by identifying elevated blood velocity in the common iliac vein, but this requires significant technical expertise 3, 4. As a result, an MTS diagnosis even in patients with symptomatic DVT may be missed. To confirm the diagnosis of MTS, other imaging modalities such as CT venography, magnetic resonance imaging (MRI), or CT abdomen and pelvis with contrast may be performed 3.

Treatment of thrombosis in the setting of MTS involves catheter-delivered thrombolytics and percutaneous mechanical thrombectomy, either with or without angioplasty and stent placement 1. Due to the pulsatile nature of mechanical arterial obstruction, MTS responds poorly to conservative management with anticoagulation alone 1. Failure to treat MTS quickly could lead to post-thrombotic syndrome (PTS), which is thought to occur as a result of valvular incompetence and venous hypertension and leads to leg swelling and chronic skin changes such as hyperpigmentation, induration, and ulceration 5. Meta-analysis has demonstrated the superiority of catheter-directed thrombolysis when compared to anticoagulation alone for the prevention and treatment of PTS 6.

Our case demonstrates how POCUS can help identify extensive DVT in an individual without classic signs of DVT. POCUS enables clinicians to visualize the anatomy and pathology of the groin, including the hip, anterior hip musculature, the inguinal lymph nodes, and inguinal hernias, which can be seen with significant sensitivity and specificity 7. Of note, POCUS can assist with identification of lower extremity proximal DVT and as such it is now routinely used in the ED, ICU, and other healthcare settings 8. Studies and meta-analyses which use POCUS to evaluate for DVT have an estimated sensitivity and specificity in the 90-100% range 8. As such, POCUS is a strong clinical tool that ought to be used more frequently by clinicians.

For our case who presented without typical findings and risk factors for DVT, the use of POCUS completely altered our differential diagnosis and led to prompt identification and treatment of MTS. The ease of access to POCUS and its efficacy at identifying extensive DVT was instrumental in the proper care of this patient. 

## Conclusion

Physicians should consider lower extremity DVT in groin-related presentations, even without classic signs of DVT. Point-of-care ultrasound is an easily accessible and can be a first-line screening tool in the identification of extensive DVT. 

## Conflict of interest

none

## Funding

none

## Patient consent

written

## Supplementary Material

Video S1 A distended, non-compressible left CFV and FV seen on point-of-care ultrasound.
